# Neurofilament Light Protein as a Potential Blood Biomarker for Huntington's Disease in Children

**DOI:** 10.1002/mds.29027

**Published:** 2022-04-18

**Authors:** Lauren M. Byrne, Jordan L. Schultz, Filipe B. Rodrigues, Ellen van der Plas, Douglas Langbehn, Peggy C. Nopoulos, Edward J. Wild

**Affiliations:** ^1^ Huntington's Disease Centre, UCL Queen Square Institute of Neurology University College London London United Kingdom; ^2^ Department of Psychiatry Carver College of Medicine at the University of Iowa Iowa City Iowa USA; ^3^ Department of Neurology Carver College of Medicine at the University of Iowa Iowa City Iowa USA

**Keywords:** biomarkers; children; Huntington's disease

## Abstract

**Background:**

Juvenile‐onset Huntington's disease (JOHD) is a rare and particularly devastating form of Huntington's disease (HD) for which clinical diagnosis is challenging and robust outcome measures are lacking. Neurofilament light protein (NfL) in plasma has emerged as a prognostic biomarker for adult‐onset HD.

**Methods:**

We performed a retrospective analysis of samples and data collected between 2009 and 2020 from the Kids‐HD and Kids‐JHD studies. Plasma samples from children and young adults with JOHD, premanifest HD (preHD) mutation carriers, and age‐matched controls were used to quantify plasma NfL concentrations using ultrasensitive immunoassay.

**Results:**

We report elevated plasma NfL concentrations in JOHD and premanifest HD mutation‐carrying children. In pediatric HD mutation carriers who were within 20 years of their predicted onset and patients with JOHD, plasma NfL level was associated with caudate and putamen volumes.

**Conclusions:**

Quantifying plasma NfL concentration may assist clinical diagnosis and therapeutic trial design in the pediatric population. © 2022 The Authors. *Movement Disorders* published by Wiley Periodicals LLC on behalf of International Parkinson Movement Disorder Society.

## Graphical abstract

Plasma NfL concentrations are significantly elevated in patients with juvenile‐onset Huntington's disease (JOHD) and children with the gene mutation that causes Huntington's disease (HD) who are 20 years from their predicted motor onset of HD. Plasma NfL levels may assist clinical diagnosis and therapeutic trial design in this pediatric population

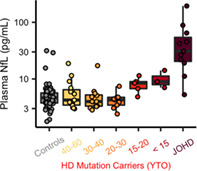

Huntington's disease (HD) is a neurodegenerative disease caused by a CAG repeat expansion within the *HTT* exon 1 that is negatively associated with the age of symptom onset. Pediatric patients have largely been excluded from HD research, creating two key knowledge gaps. First, it is unclear how early HD disease‐modifying therapies could be safely initiated, with early intervention likely to optimize preventative outcomes.^1^ However, because the huntingtin protein is important for neurodevelopment, it is necessary to distinguish between neurodevelopment and the onset of neurodegeneration.^2^, ^3^ Second, little research has been done in juvenile‐onset HD (JOHD), a rare form of HD characterized by exceptionally long CAG repeats and motor manifestation before the age of 21.^4^, ^5^ Initial manifestations of JOHD often overlap with normal variability in childhood or adolescence or with prevalent juvenile disorders such as depression, anxiety, attention deficit hyperactivity disorder (ADHD), and Tourette's syndrome, complicating difficult decisions about diagnosis and genetic testing of minors.^4^ An indicator that could distinguish a neurodegenerative process from a neurodevelopmental disorder would be a useful screening tool to inform difficult decisions on genetically testing minors. Plasma neurofilament light protein (NfL) is an established biomarker of neurodegeneration and an emerging biomarker for adult‐onset HD (AOHD) progression.^6^, ^7^, ^8^, ^9^ NfL concentrations have not been quantified previously in a pediatric HD cohort.

We quantified plasma NfL levels in two unique pediatric patient populations: healthy children with *HTT* expansion mutations expected to produce adult‐onset disease (premanifest Huntington's disease [preHD]) and those with JOHD. We compared these to NfL in healthy control children and young adults to better understand its use to monitor disease and advance clinical trial efforts in these patient populations.

## Patients and Methods

### Participants

We performed a retrospective analysis of prospectively collected data from the Kids‐HD/JHD observational studies. Kids‐HD recruited children and young adults with a parent/grandparent with a CAG expansion and healthy controls with no known family history of HD. For research purposes only, participants were genotyped in a blinded manner such that neither the children, their families, the clinicians, nor the patient‐facing researchers were aware of the test results (Appendix S1).^10^, ^11^ Those with CAG repeats ≥36 were labeled Gene‐Expanded and those with repeats less than 36, including all of the healthy control volunteers, as Gene‐Non‐Expanded (GNE). The Kids‐JHD study recruited patients with a motor diagnosis of JOHD by a neurologist before age 21 years and had a genetic diagnosis confirming HD. Both studies implemented an accelerated longitudinal design where some participants had multiple visits at 1‐ to 2‐year intervals, and others had only one visit.^10^, ^11^

Plasma NfL concentration was quantified using the Quanterix NF‐Light assay kit on the HD‐1 Simoa analyzer (see Appendix S1). We combined plasma NfL data from both Kids‐HD/JHD with previously published plasma NfL data from the longitudinal HD‐CSF cohort^8^, ^12^ (from premanifest and manifest AOHD) to assess plasma NfL trajectories over the course of HD.

### Statistical Analysis

Plasma NfL concentrations were natural‐log‐transformed to account for right‐skewed distribution.^7^ We first examined participants from Kids‐HD with premanifest AOHD (preHD). PreHD were grouped on their predicted years to onset (YTO; based on Langbehn formula^13^) and compared to GNE. Plasma NfL concentrations were compared between JOHD and GNE. For groupwise analyses, we constructed linear mixed effects regression (LMER) models controlling for age and included random effects per participant and family to account for siblings. Within‐subject and residual variances were estimated separately for groups via iteratively re‐weighted least squares (SAS v9.4). We created a receiver operating curve to determine the sensitivity and specificity of plasma NfL levels to distinguish between JOHD and GNE.

The relationship between plasma NfL measurements and striatal volume (see Appendix S1) among select preHD participants and JOHD was evaluated. Brain volumes were presented as percentage of intracranial volume (ICV), and scanner was included as a covariate in LMER models. Plasma NfL concentration versus brain models were fit using the package lmerTest (version 3.1‐2) within R (version 3.6.0).

We pooled plasma NfL measurement data from the Kids‐HD/JHD with adult participants from HD‐CSF^6^, ^8^, ^12^ to evaluate the nonlinear plasma NfL dynamics by disease burden score (DBS = age × [CAG‐35.5])^14^ using LMER models. A two degrees‐of‐freedom test was performed for the joint significance of the two‐spline transformation of DBS.

We accounted for multiple comparisons using the false discovery rate (FDR) correction when preHD groups were compared to controls. An FDR threshold of < 0.05 and a *P* < 0.05 were considered statistically significant.

### Study Approval and Informed Consent

The Kids‐HD and Kids‐JHD protocols were approved by the Institutional Review Board at the University of Iowa. The parents or legal guardians of participants who were aged below 18 years or who were above 18 with significant cognitive deficits provided written informed consent, and the children provided assent. Participants who were aged 18 years or above provided written consent. Ethical approval for HD‐CSF was provided by the London Camberwell St Giles Research Ethics Committee. All participants provided informed written consent before enrollment.

### Data Sharing

The Kids‐HD and Kids‐JHD data sets, including deidentified participant data, processed brain volumes, and clinical assessments may be made available on reasonable request.

## Results

The characteristics of the cohort are provided in Table 1. The characteristics by estimated years to motor onset (YTO) groupings are provided in Table S1. We assessed potential confounding demographics on plasma NfL levels in healthy controls, finding little evidence for the impact of healthy development on plasma NfL concentrations (Fig. S1).

**TABLE 1:** Baseline cohort characteristics of Kids‐HD and Kids‐JHD participants

	Controls	preHD	JOHD	*P*‐value
N (total visits)	61 (83)	30 (44)	9 (11)	NA
Age, mean ± SD	12.75 ± 3.71	14.00 ± 3.12	16.48 ± 6.38	0.019
Males, N (%)	23 (37.7)	10 (33.3)	5 (55.6)	0.483
CAG, mean ± SD	20.33 ± 4.39	43.90 ± 4.47	72.11 ± 12.67	<0.0001
BMI, mean ± SD	22.14 ± 7.48	21.92 ± 5.13	21.51 ± 6.22	0.963
Tanner stage, N (%)				0.337
0	15 (22.2)	4 (13.3)	2 (22.2)	
1	4 (6.6)	0 (0)	0 (0)	
2	4 (6.6)	1 (3.3)	0 (0)	
3	12 (19.7)	5 (16.7)	0 (0)	
4	16 (26.2)	15 (50.0)	4 (44.4)	
5	10 (16.4)	5 (16.7)	3 (33.3)	
Parental SES, N (%)				0.062
1	0 (0)	0 (0)	0 (0)	
2	36 (59.0)	14 (46.7)	2 (22.2)	
3	22 (36.1)	14 (46.7)	4 (44.4)	
4	2 (3.3)	2 (6.7)	2 (22.2)	
5	1 (1.6)	0 (0)	1 (11.1)	
Plasma NfL (pg/mL), mean ± SD	5.46 ± 4.78	5.67 ± 3.32	56.01 ± 58.02	<0.000

*P*‐values for continuous variables were generated from one‐way analyses of variance. *P*‐values for categorical variables were generated from Pearson's χ^2^ analyses. Values are presented as mean ± SD unless otherwise stated. Tanner stage assesses puberty stage, and parental SES was quantified using the Hollingshead Scale.

Abbreviations: HD, Huntington's disease; JOHD, juvenile‐onset Huntington's disease; NA, not available; SD, standard deviation; preHD, premanifest Huntington's disease; JOHD, juvenile‐onset Huntington's disease; CAG, cytosine‐adenine‐guanine; BMI, body mass index; SES, socioeconomic status; NfL, neurofilament light protein.

**FIG 1:**
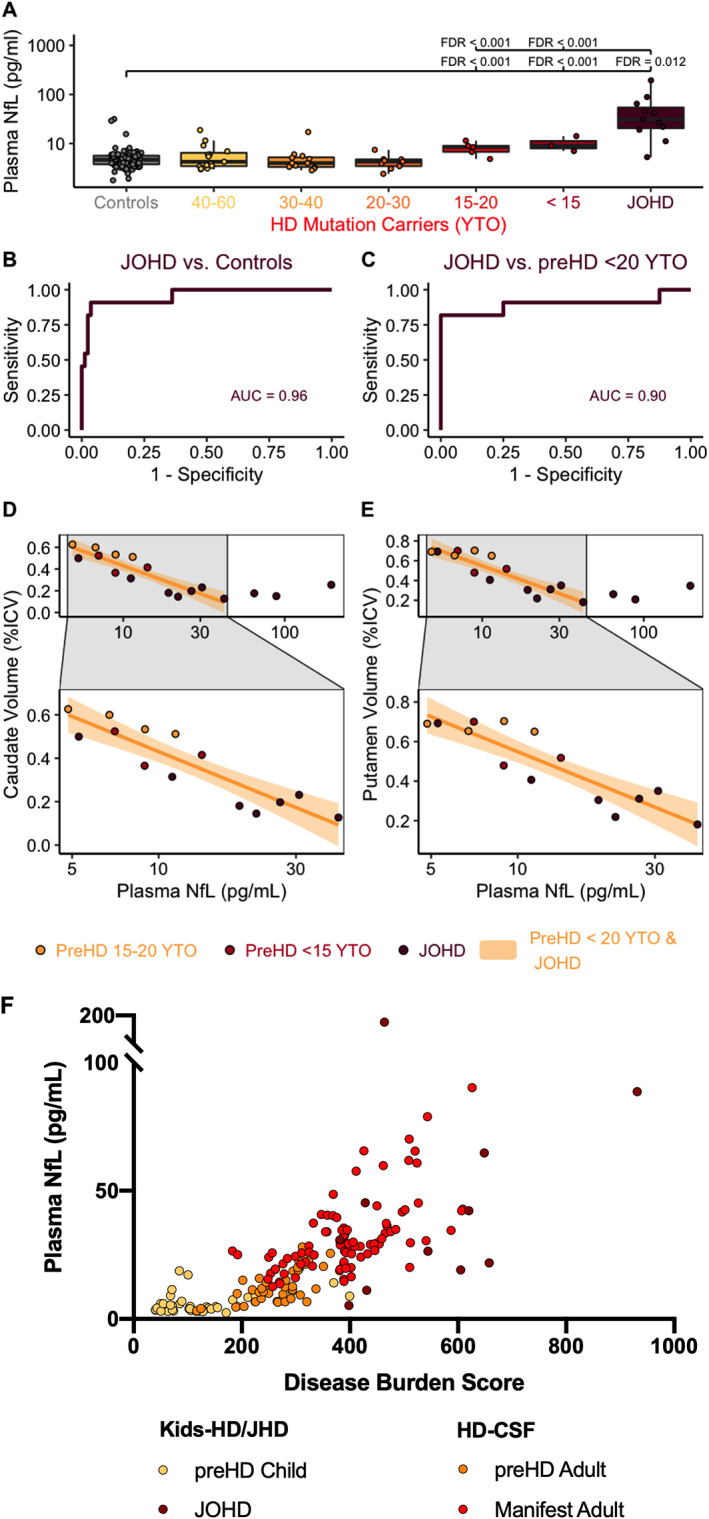
Plasma NfL is elevated in JOHD and children within 20 YTO of AOHD. Plasma NfL was elevated in (**A**) preHD children within 20 years of their predicted onset and (**B**) the JOHD group. Plasma NfL identifies (**C**) patients with JOHD. Plasma NfL in patients with JOHD and preHD participants who are close to onset is related to (**D**) caudate and (**E**) putamen volume. Plasma NfL is significantly related to disease burden in (**F**) children and adults with HD. AUC, area under the curve; HD, Huntington's disease; ICV, intracranial volume; JOHD, juvenile‐onset Huntington's disease; LMER, linear mixed effects regression model; MD, mean difference between groups; pg/mL, picograms per milliliter; NfL, neurofilament light protein; preHD, premotor‐manifest Huntington's disease; q, false discovery rate threshold; YTO, years to predicted onset of Huntington's disease. [Color figure can be viewed at wileyonlinelibrary.com]

The mean plasma NfL concentration in controls was 4.09 pg/mL (95% confidence interval [95% CI] 2.89–5.28), which did not differ significantly from preHD 40 to 60 YTO (mean difference [MD] = 0.29 pg/mL [−1.05–1.63]), 30 to 40 YTO (MD = −0.07 pg/mL [−1.43–1.29]), or 20 to 30 YTO (MD = −0.39 pg/mL [−1.68–0.89]). However, the preHD 15 to 20 YTO and <15 YTO groups both had higher plasma NfL concentrations than controls (MD = 2.39 pg/mL [1.10–3.69], FDR < 0.0001 and MD = 5.00 [3.13–6.87], FDR = 0.012, respectively; Fig. 1A).

JOHD patients had a mean plasma NfL concentration of 30.27 pg/mL (95% CI [28.46–32.07]) that was significantly elevated relative to controls (MD = 26.00 pg/mL, 95% CI [24.19–27.81], FDR < 0.0001; Fig. 1A). The mean plasma NfL concentration of the JOHD group was also significantly elevated relative to all the preHD groups, most notably the participants who were 15 to 20 YTO (MD = 23.61 pg/mL, 95% CI [21.80–25.42], FDR < 0.0001; Fig. 1A) and <15 YTO (MD = 21.00 pg/mL, 95% CI [19.19–22.81], FDR < 0.0001; Fig. 1A).

A cutoff of 9.64 pg/mL accurately classified 91% (95% CI [73%–100%]) of JOHD patients and 96% (95% CI [92%–100%]) of controls (AUC 0.96; Fig. 1B). In a post hoc analysis, we determined that a cutoff of 19.09 pg/mL accurately classified 82% (95% CI [60%–100%]) of JOHD patients and 100% (95% CI [100%–100%]) of preHD participants who were within 20 YTO (AUC: 0.90; Fig. 1C).

Among participants with elevated NfL concentrations (preHD within 20 YTO and JOHD groups combined), plasma NfL levels were significantly associated with caudate volume (β = −0.12% ICV per log pg/mL, 95% CI [−0.18 to −0.05], FDR = 0.004; Fig. 1D) and putamen volume (β = −0.14% ICV per log pg/mL 95% CI [−0.21 to −0.07], FDR = 0.004; Fig. 1E). However, the relationships seemed to disappear at NfL concentrations >50 pg/mL. Restricting the models to concentrations <50 pg/mL, the reestimated slopes were −0.18% ICV per log pg/mL for caudate (95% CI [−0.27 to −0.09], FDR = 0.004; Fig. 1D) and −0.21% ICV per log pg/mL for putamen (95% CI [−0.31 to −0.12], q = 0.004; Fig. 1E).

There was a significant, nonlinear relationship between plasma NfL level and DBS in participants from the Kids‐HD, Kids‐JHD, and HD‐CSF (an AOHD cohort) cohorts (*F*(2, 107.85) = 33.21, *P* < 0.001; Fig. 1F), with an increase in plasma NfL concentration beginning to emerge around DBS 200.

## Discussion

We report two novel findings from unique pediatric HD populations. First, plasma NfL concentration was significantly increased in children by approximately 20 years before the predicted motor onset of AOHD. Second, patients with JOHD had significantly higher plasma NfL levels than healthy controls, by a factor of about sevenfold. In those with elevated NfL concentrations up to 50 pg/mL, plasma NfL levels were significantly associated with decreasing volumes of the caudate and putamen. The relationship between striatal volume and plasma NfL concentration seemed to diminish in participants with higher NfL concentrations. Plasma NfL levels increased significantly with disease burden.

These preHD AOHD findings are consistent with the HD Young Adult study (HD‐YAS), where adult subjects about 20 years from onset had increased plasma NfL concentrations.^15^ However, this had not been previously shown in children, where normal maturational processes result in decreasing striatal volume beginning near puberty, making it difficult to distinguish when normal development ends and early degeneration begins.^10^ Plasma NfL levels could, however, help distinguish between neurodevelopmental and neurodegenerative processes. NfL concentrations may, in the future, help guide decisions around the timing of disease‐modifying interventions.

The substantial increases in plasma NfL levels observed in JOHD participants could also assist clinicians in providing a timely diagnosis to patients with JOHD. Currently, genetic testing of a minor is performed only when a provider is confident that the clinical symptoms are consistent with JOHD, which could take years. Our results demonstrate that plasma NfL concentrations can classify patients with JOHD from non‐HD controls and asymptomatic preHD children with a fairly high degree of accuracy. Therefore, plasma NfL levels could provide additional information for practitioners struggling to decide if confirmatory genetic testing is warranted in a minor when a clinical diagnosis of JOHD is not clear. Further, plasma NfL concentration could be used as a much‐needed outcome measure to facilitate therapeutics trials in JOHD populations.

There are important limitations to this work. The prevalence of disorders such as Tourette's syndrome and ADHD was low in the control group. Therefore, it is possible that a higher prevalance of these common juvenile disorders would make it more difficult to distinguish JOHD participants from controls. In addition, it is unknown if plasma NfL concentrations are elevated in other neurologic disorders that may impact children, such as dystonia or parkinsonism. Consequently, elevated NfL concentrations may lack the specificity required to distinguish between JOHD and other neurological conditions.

Collectively, these findings suggest two potential applications of plasma NfL: (1) determining the timing of intervention for young preHD subjects and (2) a marker for disease diagnosis and monitoring progression in JOHD.

## Full financial disclosures for the previous 12 months

L.M.B., F.B.R., and E.J.W. are full‐time UCL employees. L.M.B. reports salary support from the Huntington's Disease Society of America and has provided consultancy for F. Hoffmann‐La Roche AG, Genentech, Novartis International AG, Annexon Inc., Remix Therapeutics Inc., Guidepoint Global and GLG Group. J.L.S. receives salary support from the National Center for Advancing Translational Sciences (NIH KL2TR002536) and The Michael J. Fox Foundation for Parkinson's Research. F.B.R. reports salary support from the CHDI Foundation and has provided consultancy to GLG and Roche. E.J.W. reports grants from the Medical Research Council UK, the CHDI Foundation, and F. Hoffmann‐La Roche Ltd. during the conduct of the study and personal fees from F. Hoffman‐La Roche Ltd., Triplet Therapeutics, PTC Therapeutics, Shire Therapeutics, Wave Life Sciences, Mitoconix, Takeda Pharmaceuticals Ltd., and Loqus23. All honoraria for these consultancies were paid through the offices of UCL Consultants Ltd., a wholly owned subsidiary of University College London. University College London Hospitals NHS Foundation Trust has received funds as compensation for conducting clinical trials for Ionis Pharmaceuticals, Pfizer, and Teva Pharmaceuticals. D.L. receives research funding from the National Institute of Neurological Disorders and Stroke, the CHDI Foundation, University College London, and from the Wellcome Trust. During the past 12 months, D.L. has served as a paid statistical consultant for the design of Huntington's disease trials for Novartis AG, Voyager Therapeutics, Spark Therapeutics, and Takeda Pharmaceutical Company Limited. He has also been a paid consultant to Trinity Life Sciences. P.C.N. and E.P. report no competing interests.

## Author Roles

- L.M.B.: study design, execution, analysis, writing, editing of the final version of the manuscript.
- J.L.S.: study design, execution, analysis, writing, and editing of the final version of the manuscript.
- F.B.R.: study execution, analysis, writing, and editing of the final version of the manuscript.
- E.P.: analysis and editing of the final version of the manuscript.
- D.L.: study design, analysis, and editing of the final version of the manuscript.
- P.C.N.: study design, execution, analysis, and editing of the final version of the manuscript.
- E.J.W.: study design, execution, analysis, and editing of the final version of the manuscript.
- L.M.B. and J.L.S. share first author position, and P.C.N. and E.J.W. share senior author position, with the authorship order assigned alphabetically.

## Supporting information


**APPENDIX S1.** Supporting Information

## Data Availability

The Kids‐HD and Kids‐JHD datasets, including deidentified participant data, processed brain volumes, and clinical assessments may be made available upon reasonable request.
