# Supplementary material for: Neurofilament Light Protein as a Potential Blood Biomarker for Huntington's Disease in Children
Source: Mov Disord. 2022 Apr 18;37(7):1526–31. doi: 10.1002/mds.29027 (PMC9308659; doi:10.1002/mds.29027)
Supplement: Supplementary file 1 — APPENDIX S1. Supporting Information [file MDS-37-1526-s001.docx]

**Supplemental Material**

**eMethods**

*Genetic Testing of Minors for Research Purposes Only:* All participants in the Kids-HD/JHD studies provided either saliva or blood for genetic testing. Genetic testing was done for research purposes only; results were not revealed to participants, their families, clinicians, or researchers. This was true of participants in the Kids-JHD study who had already undergone genetic testing. Genetic testing results were sent to a clinical research team member who was not involved in any study visits. This team member deidentified all results to ensure all team members who directly interacted with participants were blinded to genetic testing results. Full study protocols for Kids-HD/JHD studies are included as supplement 2 and 3 respectively.

## *Plasma Sample Collection and Storage:* Kids-HD/JHD blood samples were collected in EDTA Vacutainer tubes (BD). All samples were coded with a unique study ID and were sent to the University of Iowa Diagnostic Laboratories. Samples were briefly stored on ice and then centrifuged at 2,000g for x minutes. Plasma was decanted before being centrifuged at 3,000g at 4°C for 15 minutes then pipetted into 1.2-mL cryotubes in 500 uL aliquots and frozen and stored at -80°C before being shipped.

## *NfL Quantification:* Kids-HD/JHD plasma NfL concentrations were measured using the commercially available NF-Light^®^ assay kit from on the Simoa HD-1 analyzer platform as per the manufacturer’s instructions (Quanterix, Lexington). NfL was quantified in duplicate for all plasma samples. The intra-assay coefficient of variation (CV) was 6.5% and inter-assay CV was 1.65%. HD-CSF plasma NfL was previously quantified and published as per Rodrigues et al.^6^

## *Image Acquisition and processing:* The structural imaging acquisition methods have been described previously.^10^ A 3T Siemens Trio TIM (Siemens AG, Munich, Germany) machine was used to acquire images prior to June 2016 (n=33). After June 2016, a 3T General Electric Discovery MR750w (GE Medical Systems, Chicago, IL) was used (n=17). Full details regarding image processing have been previously described by van der Plas, et al.^10^

*Supplemental* Table 1: Baseline Cohort Characteristics by Years to Onset Groupings

|  | Controls | 40-60 YTO | 30-40 YTO | 20-30 YTO | 15-20 YTO | <15 YTO | P-Value |
| --- | --- | --- | --- | --- | --- | --- | --- |
| N (total visits) | 61 (83) | 9 (15) | 10 (12) | 7 (9) | 2 (4) | 2 (3) | NA |
| Age,  mean ± S.D. | 12.75 ± 3.71 | 13.99 ± 2.07 | 13.47 ± 3.93 | 13.33 ± 2.98 | 15.04 ± 2.65 | 17.96 ± 3.12 | 0.355 |
| Female,  N (%) | 23 (37.7) | 4 (44.4) | 8 (80.0) | 6 (85.7) | 1 (50.0) | 1 (50.00) | 0.488 |
| CAG,  mean ± S.D. | 20.33 ± 4.39 | 39.78 ± 0.97 | 42.60 ± 0.84 | 46.71 ± 2.29 | 49.00 ± 1.41 | 54.00 ± 7.07 | <0.0001 |
| YTO,  mean ± S.D. | NA | 50.39 ± 6.23 | 36.38 ± 2.03 | 24.39 ± 2.87 | 17.96 ± 0.79 | 10.39 ± 1.93 | <0.0001 |
| BMI,  mean ± S.D. | 22.14 ± 7.48 | 22.63 ± 5.88 | 20.68 ± 4.83 | 21.99 ± 4.86 | 18.23 ± 1.16 | 28.42 ± 1.30 | 0.726 |
| Tanner Stage, N (%)  0  1  2  3  4  5 | 15 (22.2)  4 (6.6)  4 (6.6)  12 (19.7)  16 (26.2)  10 (16.4) | 0 (0.0)  0 (0.0)  1 (11.1)  4 (44.4)  4 (44.4)  0 (0.0) | 3 (30.0)  0 (0.0)  0 (0.0)  0 (0.0)  5 (50.0)  2 (20.0) | 1 (14.3)  0 (0.0)  0 (0.0)  0 (0.0)  4 (57.1)  2 (28.6) | 0 (0.0)  0 (0.0)  0 (0.0)  1 (50.0)  1 (50.0)  0 (0.0) | 0 (0.0)  0 (0.0)  0 (0.0)  0 (0.0)  1 (50.0)  1 (50.0) | 0.598 |
| Parental SES, N (%)  2  3  4  5 | 36 (59.0)  22 (36.1)  2 (3.3)  1 (1.6) | 5 (55.6)  3 (33.3)  1 (11.1)  0 (0.0) | 7 (70.0)  3 (30.0)  0 (0.0)  0 (0.0) | 1 (14.3)  5 (71.4)  1 (14.3)  0 (0.0) | 1 (50.0)  1 (50.0)  0 (0.0)  0 (0.0) | 0 (0.0)  2 (100.0)  0 (0.0)  0 (0.0) | 0.696 |
| Plasma NfL,  mean ± S.D. | 5.46 ± 4.78 | 5.30 ± 2.71 | 5.59 ± 4.20 | 4.59 ± 1.41 | 6.65 ± 2.55 | 10.58 ± 5.01 | 0.677 |
| P-values for continuous variables were generated from one-way analyses of variance. P-values for categorical variables were generated from Pearson Chi-Square analyses. Values are presented as mean ± SD unless otherwise stated.  SD, Standard deviation; CAG, Cytosine-Adenine-Guanine; YTO, years to onset; NA, Not applicable; BMI, Body mass index; SES, socioeconomic status, measured by the Hollingshead Scale; NfL, neurofilament light protein. | | | | | | | |


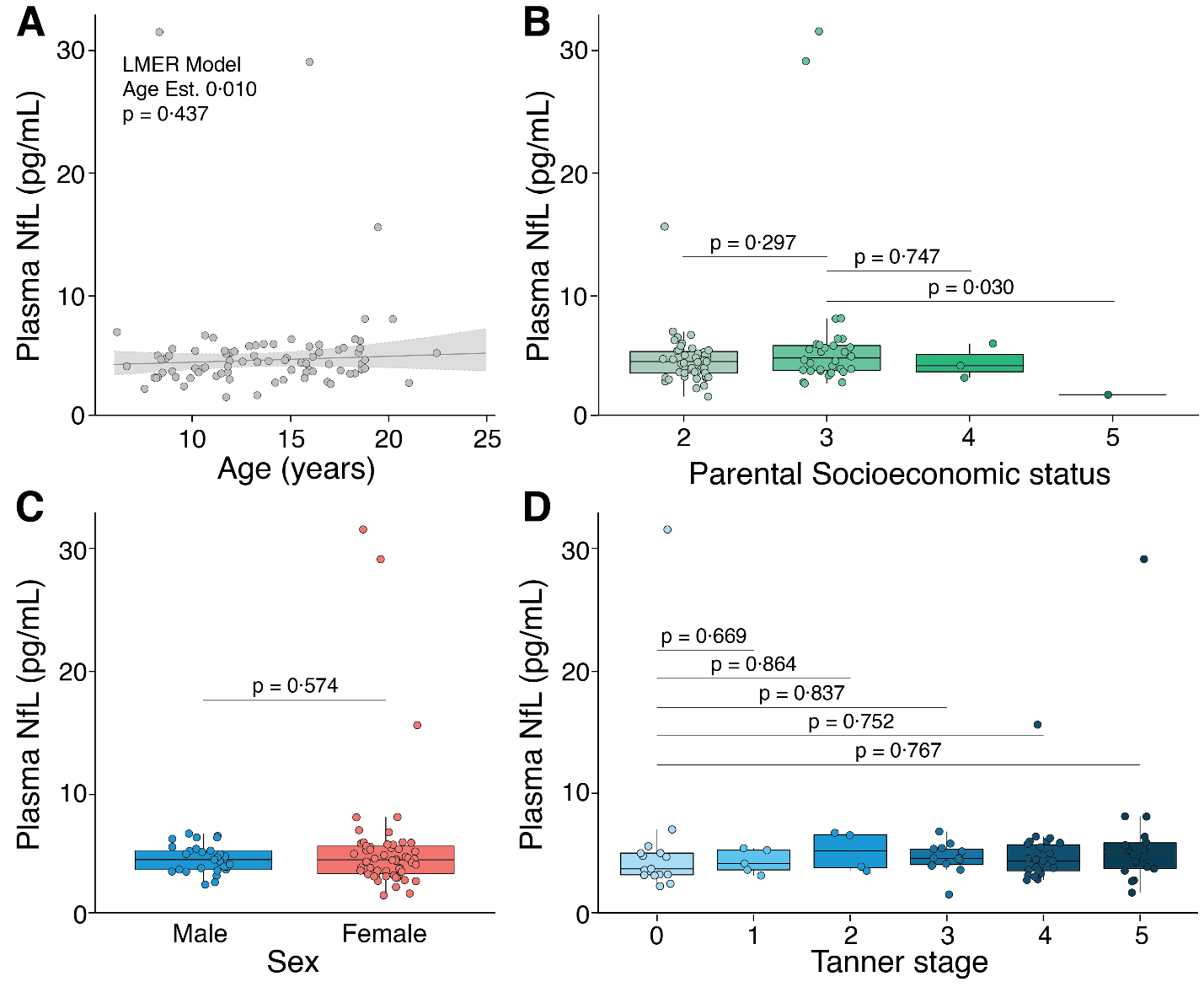


Supplemental Figure 1: Plasma NfL in healthy development and potential confounding demographics.

Plasma NfL concentrations are plotted against a, age b, parental socioeconomic status c, sex and d, Tanner stage (puberty) in healthy control individuals (n=61). Data was available from multiple timepoints for some individuals (83 observations). P-values were generated from linear mixed effects regression models with a random participant effect (intercept). Pairwise tests were post-hoc t-tests using Satterthwaite's method. Raw NfL values are presented. Models used natural log transformed NfL data and was back-transformed for a, the age model. NfL, neurofilament light protein; LMER, linear mixed effects regression.
